# Individual and contextual risk factors for chikungunya virus infection: the SEROCHIK cross-sectional population-based study

**DOI:** 10.1017/S0950268818000341

**Published:** 2018-05-03

**Authors:** A. Fred, A. Fianu, M. Béral, V. Guernier, D. Sissoko, M. Méchain, A. Michault, V. Boisson, B.-A. Gaüzère, F. Favier, D. Malvy, P. Gérardin, SEROCHIK group

**Affiliations:** 1 INSERM CIC1410, CHU Réunion, Saint Pierre, Reunion, France; 2 Department of Social and Preventive Medicine, School of Public Health, Montreal University, Montreal, Canada; 3 UMR 1309 CMAEE ‘Contrôle des maladies animales, exotiques et émergentes’, CIRAD, Sainte Clotilde, Reunion, France; 4 Ministère de l'Agriculture, Direction Régionale de l'Agriculture et de la Forêt, Dijon, France; 5 Université de La Réunion, CRVOI ‘Centre de Recherche et de Veille de l'océan Indien’, CYROI, Sainte Clotilde, Reunion, France; 6 Australian Institute of Tropical Health and Medicine, James Cook University, Townsville, Queensland, Australia; 7 Department of Tropical Medicine and Clinical International Health, CHU Bordeaux, Bordeaux, France; 8 Infectious Diseases in Low Income Countries (IDLIC), Bordeaux Population Health Research Centre (INSERM U1219, Université de Bordeaux, ISPED), Bordeaux, France; 9 Bacteriology, Virology and Parasitology lab, CHU Réunion, Saint Pierre, Reunion, France; 10 Polyvalent Intensive Care Unit, CHU Réunion, Saint Pierre, Reunion, France; 11 Polyvalent Intensive Care Unit, CHU Réunion, Saint Denis, Reunion, France; 12 Université de La Réunion, CNRS 9192, INSERM U1187, IRD 249, CHU Reunion, UM 134 PIMIT ‘Processus Infectieux en Milieu Tropical’, CYROI, Sainte Clotilde, Reunion, France

**Keywords:** Chikungunya, cross-sectional study, disease burden, population attributable fraction, risk factor

## Abstract

The purpose of the study was to weigh the community burden of chikungunya determinants on Reunion island. Risk factors were investigated within a subset of 2101 adult persons from a population-based cross-sectional serosurvey, using Poisson regression models for dichotomous outcomes. Design-based risk ratios and population attributable fractions (PAF) were generated distinguishing individual and contextual (i.e. that affect individuals collectively) determinants. The disease burden attributable to contextual determinants was twice that of individual determinants (overall PAF value 89.5% *vs*. 44.1%). In a model regrouping both categories of determinants, the independent risk factors were by decreasing PAF values: an interaction term between the reporting of a chikungunya history in the neighbourhood and individual house (PAF 45.9%), a maximal temperature of the month preceding the infection higher than 28.5 °C (PAF 25.7%), a socio-economically disadvantaged neighbourhood (PAF 19.0%), altitude of dwelling (PAF 13.1%), cumulated rainfalls of the month preceding the infection higher than 65 mm (PAF 12.6%), occupational inactivity (PAF 11.6%), poor knowledge on chikungunya transmission (PAF 7.3%) and obesity/overweight (PAF 5.2%). Taken together, these covariates and their underlying causative factors uncovered 80.8% of chikungunya at population level. Our findings lend support to a major role of contextual risk factors in chikungunya virus outbreaks.

## Introduction

Chikungunya virus (CHIKV) is responsible for chikungunya fever (CHIKF), a dengue-like illness characterised by persistent incapacitating polyarthralgia [1,2]. Between its first description in Tanzania in the early 50s [3], and its re-emergence in Lamu island (Kenya) in 2004 and subsequent spread in the Indian ocean in 2005–2006 [4], CHIKF was regarded as a rather harmless neglected tropical disease limited to developing countries. Since the occurrence of atypical and severe forms of CHIKF [5–8], and its more recent spread to Europe [9] and to the Americas [10], CHIKV has increasingly been recognised as a leading threat for public health, through the global expansion of its mosquito vectors, *Aedes (Ae) albopictus* and *Ae aegypti*.

Between March 2005 and August 2006, Reunion island experienced a major outbreak of CHIKF [11] due to the circulation of a new genotype of the African variant of the virus, renamed Indian ocean lineage, together with the permissive adaption of the local competent vector, *Ae albopictus* [12]. At the end of the epidemic, a population-based serosurvey highlighted a huge penetration of the virus in the community with a burden of ~266 000 clinical cases (attack rate: ~35%) and 300 000 infected persons (prevalence rate: 38.2%) [4,11]. So far, the reasons why transmission was halted at a prevalence of ~40% remain to be elicited, as such a figure contradicts prediction of herd immunity that estimated the protective level at above 70% [13].

Indeed, the understanding of the community burden of CHIKF is a puzzling problem and constitutes a challenge for epidemiologists and public health stakeholders aiming to implement control measures in the event of future outbreaks.

So far, a few population-based cross-sectional serosurveys have proposed the investigation of CHIKF determinants in distinguishing individual and contextual risk factors [14–17]. None of these has been able to guide decision-making based on appropriate public health impact measures such as population attributable fractions (PAF). With the exception of one study [18], all previous studies have based identification of CHIKF determinants on logistic regression approach taking prevalence odds ratio, best known as the odds ratio (OR), as the ‘effect measure’ of interest [19]. These however represent only proxies of the log-binomial model and prevalence proportion ratio (PPR), the gold standard method and estimator in cross-sectional studies, because the OR often tends to overstate the PPR when the outcome is common [19], and hence the OR is more difficult to interpret (i.e. the former can be interpreted only in terms of strength of association, while the latter is multiplicative and can be interpreted in terms of multiplying/dividing the prevalence estimates) [20]. In this context, given the propensity of the OR to overestimate the PPR [21], we chose the Poisson regression model and the incidence rate ratio (IRR), as two proxies of the log-binomial model and PPR, respectively. We also used PAF, free of hypotheses on causation, with the aim of uncovering risk patterns hosting the determinants of increased CHIKV susceptibility to be investigated in further study of causal pathways.

The objective of the present study was to weigh the community burden of CHIKF risk factors using IRR and related PAF. Determinants were identified to be from primarily individual or contextual origin, and investigated separately before being pooled. This purpose was chosen to guide further prioritisation of the most effective public health interventions for mitigating future outbreaks, based on amendable determinants.

## Methods

The SEROCHIK cross-sectional serosurvey was conducted between 17 August and 20 October 2006, shortly after the end of the chikungunya epidemic on Reunion island.

A brief description of the study setting is displayed in Supplementary Appendix 1.

### Survey design and procedures

The number of subjects needed to obtain a representative random sample of the general population was estimated to be 2640, assuming 35 ± 2% expected prevalence [4], an *α* risk of 5%, 20% proportion of refusal or absenteeism and a cluster effect.

The sample was built using a two-level probability sampling procedure. At level 1, a random draw for households (primary sampling unit) was carried out. Selection was conducted in six putative strata defined by the crossed combinations of the dwelling type (individual/collective 2–20 units/collective >20 units) and size of municipality (⩽10 000/>10 000 inhabitants). Overall, five strata were obtained after removing an empty stratum. At level 2, following a predefined Kish method [22], the field investigator randomly assigned one ‘index person’ (the Kish individual) per household to be interviewed within the selected dwellings. In accordance, a sampling fraction ranging from 0.071 to 1 was generated.

To ensure that the sample was representative of the underlying population, we corrected the sample for age, gender, dwelling and residence area based on socio-demographic information provided by the 2006 census data. Thus, a set of weights was used to increase or decrease the influence of under-/over-represented groups selected from the source population.

#### Data collection

Structured questionnaires, administered by 25 field investigators, aimed at collecting data on demographics, health, knowledge on transmission and prevention of CHIKF, dwelling and close environment of residence. These questionnaires are listed in Supplementary Appendix 2.

Individual characteristics included gender, age, occupation, chronic disease, body mass index (BMI), four items dedicated to the knowledge of CHIKV transmission (scored 0–4) and 11 preventive behaviours against *Ae albopictus* (scored 0–11).

Contextual household variables included the type and altitude of dwelling, household size, area of residence, recent history of CHIKF in the neighbourhood and a social deprivation proxy-index characterising the socio-economic status of the municipality at ecological level.

The construction of this deprivation index is detailed in Supplementary Appendix 3.

#### Outcome measure

Each participant consented to a fingertip prick and the outcome measured was the result (positive or negative) of CHIKV-specific IgG ELISA serology detected on filter paper-absorbed blood (Whatman 903^®^ Protein Saver™ Card, Schleicher & Schuell) [11]. The full procedure of blood testing is detailed in Supplementary Appendix 4.

#### Additional climate variables

After the geo-referencing of each participant's address at IRIS (‘Ilots Regroupés pour l'Information Statistique’) level, the smallest geographical unit for acquiring aggregated socio-environmental information in France, monthly meteorological data gathered from the 21 closest weather stations were obtained to complete the questionnaire data, using Margouill@ website (http://www.margouilla.net).

The climatic parameters considered were ‘cumulated rainfall’, average, maximal and minimal temperatures, and average solar radiation. The *rationale* for choosing these variables is explained in Supplementary Appendix 5.

For CHIKV-infected (CHIK+) symptomatic subjects, we inputted the average values of the closest station from the data recorded in the 2 months preceding the first CHIKF case included in the IRIS. For CHIKV-naïve (CHIK−) individuals, we used the average values of the closest station between the first and the last cases reported in the same IRIS.

### Statistical analysis

Given the subjective nature and need for intelligibility of some individual variables, comparison of outcome was restricted to people aged 15 years or more to ensure reliable data input based on personal information. First, we examined associations between CHIKF (design-based seroprevalence weighted by sampling fraction) and both individual and contextual characteristics. Design-based crude IRR and 95% confidence intervals (CI) were assessed using *χ*^2^ likelihood ratio tests weighted by sampling fraction.

Second, we investigated independent risk factors for CHIKF in a four-step multivariate Poisson regression for dichotomous outcome, modelling the fixed effects (no random intercept to allow contextual effects). In the first step, we fitted two distinct full models with respect to the intrinsic nature of the variables, individual or contextual (grouping within households) with all the relevant covariates found in bivariate analysis. In the ‘individual-variable’ model, we forced gender and age, potentially associated with unadjustable determinants to minimise residual confounding. In the second step, we fitted one unique parsimonious, ‘explicative’ model with all the putative risk factors previously identified using a manual stepwise backward elimination procedure (*P* value < 0.05 to be retained in the model). All interaction terms between the variables included in this model were tested using the Mantel–Haënszel method [23]. In the third step, we studied the contribution of climate variables. Finally, in the fourth step, we added the significant climate variables to the precedent ‘explicative’ model. All the covariates within this final ‘decision-making’ model were set as binary. At each step, we determined the pseudo-likelihood of the Poisson regression model using a survey-adjusted Wald test.

For all these analyses, our purpose was to weigh the ‘explicative’ part of each model using the combined adjusted PAF for cross-sectional data, which were produced from each specific PAF value (for overall PAF calculations, see Supplementary Appendix 6). PAF indicates the percentage of cases that would not occur in a population if the factor was eliminated. Subsequently, we gauged each key determinant of the final ‘decision-making’ model according to its amendable potential, and classified the risk factors into amendable and non-amendable, if they appeared to be causal.

All the analyses were performed using Stata14^®^ (StataCorp. 2015, College Station, TX, USA) excluding observations with missing data.

### Ethics and funding

The SEROCHIK serosurvey was approved by the ethical committee for studies with human subjects of Bordeaux (No 2006/47) and the National Commission for Informatics and Liberty, the French Data Protection Authority. All participants provided their informed consent to answer the questionnaire and for blood collection. Parent or guardian of all child participants provided informed consent on their behalf.

The study was funded by the National Institute of Health and Medical Research. The funding source had no role in study design, data collection, analysis and interpretation. The study respected the STROBE statement (Checklist in Supplementary Appendix 7).

## Results

### Study population

**Fig. 1.**
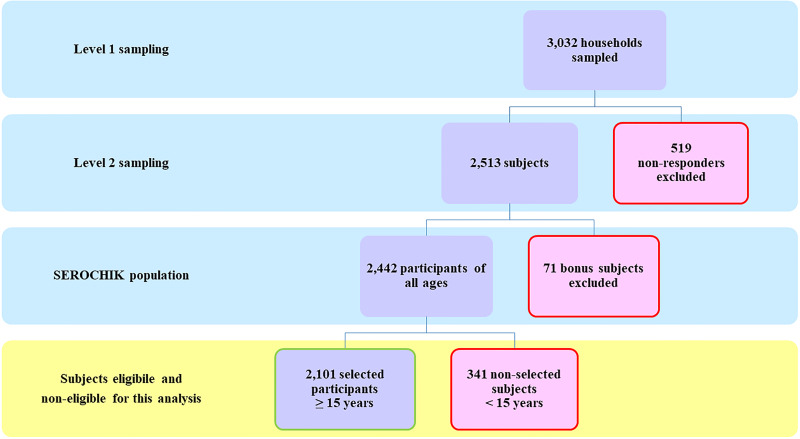
Study population. Flow chart of the study population.

The selection of the study population is presented in Figure 1. Of the 3032 subjects sampled at level 1, 2442 Kish individuals were surveyed by field investigators and 2101 participants aged 15 years or more were analysed. This sample was representative of the habitat in terms of areas of residence and altitude of dwelling place. Beyond these contextual features, it was also representative of individual characteristics such as obesity, hypertension, diabetes and asthma. Of note, our population was shifted towards the over-representation of females (59.8% *vs*. 49.3%, *P* < 0.01), elder people (⩾ 50 years, 40.1% *vs* 33.4%, *P* ⩽ 0.01) and residents of individual house (78.5% *vs* 56.4%, *P* < 0.01) (Table 1).

**Table 1.** Characteristics of the 2101 subjects (⩾15 years) analysed for chikungunya risk factors related to population structure and missing data, SEROCHIK study, August–October 2006, Reunion island

Characteristics, *n* (%)	SEROCHIK	INSEE^a^
	2101	787 836
Gender		
Women	1195 (56.9)	399 658 (49.3)^b^
Men	906 (43.1)	388 178 (50.7)
Age (years)		
15–29	484 (23.0)	107 263 (20.9)
30–39	376 (17.9)	116 600 (22.7)
40–49	398 (18.9)	118 175 (23.0)
50–59	356 (16.9)	81 147 (15.8)
60–69	244 (11.6)	48 845 (9.5)
⩾70	243 (11.6)	41 755 (8.1)
Residential area^c^		
North	499 (24.0)	185 926 (23.6)^b^
West	520 (26.6)	205 755 (26.1)
South	790 (36.9)	290 111 (36.8)
East	292 (12.5)	106 044 (13.5)
Dwelling		
Collective	412 (21.5)	343 497 (43.6)
Individual	1503 (78.5)	444 339 (56.4)

a Institut National de la Statistique et des Etudes Economiques.

b 2006 census.

c La Réunion island is divided into four administrative residential areas (microregions): North, West, South and East.

Data are given as numbers and percentages in parentheses.

### Individual determinants of chikungunya

The subjects aged 60–69 or 70 years or more, overweight (25 ⩽ BMI < 30 kg/m^2^) or obese (BMI ⩾ 30 kg/m^2^), reporting a chronic disease or no occupation were more likely to be infected (Supplementary Table S1). Among active people, farm workers (<10% of the population) were more likely to be infected (*P* = 0.01). Self-use of repellent sprays or creams (used within more than half of the population) was protective against CHIKF (*P* < 0.01). Indoor insecticide users (~40% of the population) were slightly protected compared with non-users (*P* = 0.04). Paradoxically, infection was more common in subjects reporting one or more of the following altruistic behaviours aimed at reducing the amount of breeding sites: covering tanks and water supplies (*P* = 0.01), putting sand in containers (*P* = 0.02), pruning shrubs and cleaning wastes (*P* < 0.01), removing clutter in the courtyard (*P* < 0.01). Other protective behaviours against day-biting *Ae albopictus* adult females were not associated with CHIKF (data not shown). However, when adding 11 of the 16 preventive behaviours in a score, we observed a negative correlation between the reported behaviours and exposure level, the subjects cumulating 6–11 protective behaviours being the more likely to be infected (Supplementary Table S1).

Importantly, scoring knowledge of CHIKV transmission by summing good answers to four key questions revealed higher risk for the subjects denying *Ae albopictus* as the main vector. Interestingly, this score was driven by literacy (*P* < 0.01), place of birth (*P* < 0.01) and marital status (*P* = 0.02) (Supplementary Table S2). Knowledge features showed a protective effect restricted to individuals aged <50 years (data not shown).

Finally, when adjusting eight individual covariates together (forcing gender but excluding the behaviour score), only the absence of occupation was associated with CHIKF (adjusted IRR 1.42, 95% CI 1.20–1.66), even though obesity (adjusted IRR 1.29, 95% CI 1.05–1.58) and poor knowledge on CHIKV transmission (score = 1: adjusted IRR 1.27; 95% CI 1.01–1.59; or score = 0: adjusted IRR 1.33; 95% CI 1.05–1.69) remained linked to CHIKF (Supplementary Table S3).

Taken together, the ‘individual-covariate full model’ and its underlying causative factors uncovered 44.1% of CHIKF (95% CI 41.5–46.6%). In other words, if CHIKF had been fully preventable, control measures aimed at protecting the specific groups of individuals presenting the eight abovementioned risk factors would have diminished the disease burden by more than 40%.

### Contextual determinants of chikungunya

Individual house or dwelling located under 750 m height (peak of prevalence observed in the 250–500 m stratum), or in the eastern, southern or western microregions (peak of prevalence in the eastern rainy microregion), were associated with infection (Supplementary Table S4).

Higher risks were observed in people living alone or in households of at least five persons, or reporting a CHIKF history in the neighbourhood. In addition, makeshift constructions (wooden creole house under sheet) and absence of windows screen increased exposure (*P* *<* 0.01 for both factors, respectively). At least, risk was increased in municipalities of low-to-intermediate socio-economic level (both at *P* < 0.001). When integrating climate variables in a model controlling altitude and area of residence (Supplementary Table S5 and Fig. S6), maximal temperature in the month preceding introduction of CHIKV (Tmax-1) in the IRIS was predictive of infection (adjusted IRR 1.14; 95% CI 1.10–1.19) while rainfall (Pluv-1) was very marginal (adjusted IRR 1.1001; 95% CI 1.1000–1.002).

However, when controlling eight of the abovementioned covariates, while individual house (adjusted IRR 1.67; 95% CI 1.34–2.08), CHIKF history in the neighbourhood (adjusted IRR 1.92; 95% CI 1.41–2.60), low-to-intermediate socio-economic level (intermediate: adjusted IRR 1.31; 95% CI 1.05–1.64; deprived: IRR 1.33; 95% CI 1.12–1.56), Tmax-1 (>28.5 °C: adjusted IRR 1.59; 95% CI 1.37–1.83) and Pluv-1 (>65 mm: adjusted IRR 1.31; 95% CI 1.15–1.49) remained independently associated with infection, altitude of dwelling, area of residence and household size were no longer linked to CHIKF, except for categories of people living alone (adjusted IRR 1.15; 95% CI 1.01–1.30) or below 250 m (adjusted IRR 1.94; 95% CI 1.10–3.43) (Supplementary Table S7). Adjusting on climate variables unravelled a weak negative interaction between Tmax-1 and altitude (Mantel–Haënszel OR 0.99; *P* < 0.01) that we chose to ignore in this intermediate analysis.

Taken together, the ‘contextual-covariate full model’ and its underlying causative factors uncover 89.5% of CHIKF (95% CI 83.4–93.3%). In other words, control measures aimed at protecting the specific groups of individuals living under the eight abovementioned contextual conditions would have diminished the disease burden by ~90%.

Herein, and given PAF values, contextual characteristics uncovered a greater proportion of infections (about double) than individual characteristics.

#### Influence on modelling on key determinants of chikungunya

For explicative purposes, we decided to weigh individual and contextual risk factors in a full model (data not shown). Overall, adjustment for contextual variables altered marginally the strength of individual characteristics (Table 2). Similarly, adjustment for individual characteristics ruled out the ‘household effect’ while the strength of other contextual variables was slightly modified, with the exception of altitude. Indeed, controlling both individual and contextual characteristics, without considering climate variables, exhibited a negative dose–response effect between altitude and CHIKF, with a maximal exposure below 250 m. Of note, this was observed despite a strong synergistic interaction between altitude and dwelling (Mantel–Haënszel OR 2.58; *P* = 0.01). Thus, individual house increased the risk substantially for those living in the 250–500 m stratum. As expected, combining all relevant risk factors in the ‘explicative’ model allowed the more comprehensive overview of CHIKF at community level. This was evidenced by increasing overall PAF values (Table 2), the seven covariates uncovering 92.2% (95% CI 85.7–95.7%) of infections. Importantly, this model allowed prioritisation of risk factors using PAF values, the main contributors being altitude of dwelling, then by decreasing order, history of CHIKF in the neighbourhood (hereafter designated as clustering), dwelling, socio-economic level of municipality (indicative of social deprivation), occupation, knowledge of transmission and the BMI.

**Table 2.** Multivariate explicative model of individual and contextual risk factors for chikungunya among 2101 subjects (⩾15 years), SEROCHIK survey, August–October 2006, Reunion island

Multivariate model		Poisson^a^			
Individual variables^b^	Pe_i_ (%)	aIRR	(95% CI)	PAF (%)	(95% CI)
Occupation^c^				**15.4**	**(11.4–19.2)**
Yes	57.65	1			
No	42.35	**1.37**	**(1.19–1.57)**		
Body mass index (kg/m^2^)				**6.2**	**(3.7–8.6)**
<25	57.84	1			
25–29.9	30.20	1.13	(0.98–1.30)		
⩾30	11.95	**1.29**	**(1.06–1.58)**		
Knowledge score^d^				**12.0**	**(8.6–15.1)**
Four good answers	17.86	1			
Three good answers	30.84	1.08	(0.89–1.31)		
Two good answers	21.75	1.12	(0.90–1.37)		
One good answer	16.26	**1.39**	**(1.12–1.73)**		
Zero good answer	13.29	**1.27**	**(1.00–1.60)**		
Contextual variables^e^					
Dwelling				**38.6**	**(33.0–43.7)**
Collective	11.12	1			
Individual	88.88	**1.77**	**(1.41–2.21)**		
Chikungunya history in the neighbourhood^f^				**43.7**	**(36.8–49.8)**
No	6.49	1			
Yes	73.53	**1.97**	**(1.43–2.70)**		
Unspecified	20.00	**1.61**	**(1.14–2.27)**		
Altitude of dwelling (m)				**61.2**	**(53.1–67.8)**
<250	72.68	**2.84**	**(1.52–5.28)**		
250–499	15.46	**2.47**	**(1.29–4.75)**		
500–749	7.79	**2.34**	**(1.20–4.54)**		
750–999	2.40	1.24	(0.55–2.74)		
⩾1000	1.67	1			
Socio-economic level of the municipality^g^				**16.7**	**(13.5–19.7)**
Advantaged	37.80	1			
Intermediate	24.98	**1.37**	**(1.10–1.70)**		
Deprived	37.22	**1.36**	**(1.16–1.59)**		
Overall PAF value	100	–		**92.2**	**(85.7–95.7)**

Bold characters highlight significant risk factors.

a Poisson regression model. Pe_i_: proportion of exposed individuals in the infected population are given as percentages. aIRR: adjusted incidence rate ratios and 95% CI: 95% confidence intervals. Population attributable fractions (PAF) and 95% CI are given as percentages.

b Individual variables are defined for personal (individual) exposures.

c Regular working or studying occupation.

d Score based on four questions (agree/disagree/1 point): Is chikungunya a mosquito-borne virus? Can chikungunya be transmitted by all species of mosquito? Can the mosquito transmit chikungunya to human? Can the human transmit the virus to the mosquito?

e Collective (grouping) variables are defined for contextual (household or area-level) exposures.

f Previous history of chikungunya fever in the neighbourhood indicative of clustering.

g Derived from a homemade socio-economic index categorising the 24 municipalities of the island into tree levels based on three indices: socio-economic composition (three variables), spatial segregation of ethnic minorities (one variable), existence of measures promoting social cohesion (one variable).

Given the possibility that altitude be confounded by temperature, we tested the robustness of our model by adding Tmax-1 and Pluv-1 in a final ‘decision-making’ model. Prior to this model, by pooling the participants reporting a CHIKF history in their neighbours with those for whom this information was missing, we identified the dwelling as an effect modifier of clustering (Mantel–Haënszel OR 2.33; *P* *<* 0.01), this latter being relevant in individual house. Overall, this model retained nine significant covariates plus the interaction term (Table 3). Interestingly, the hierarchy of each specific PAF value was drastically modified, and the final ‘decision-making’ model highlighted the prominence of contextual variables, while the overall PAF of the model was slightly diminished, its covariates and underlying causative factors uncovering 80.8% of CHIKF (95% CI 77.7–83.4%). Importantly, only one individual key determinant, the knowledge score, was found to be amendable, whereas three of the six contextual variables seemed relevant to guide the prioritisation of real-time control measures. Thus, we deemed that the community-based interventions around CHIKF clusters, focused on people living in individual houses within intermediate or deprived areas should be the most appropriate control measures for public health action in our context.

**Table 3.** Multivariate decision-making model of individual and contextual binary risk factors for chikungunya among 2101 subjects (⩾15 years), SEROCHIK survey, August–October 2006, Reunion island

Multivariate model			Poisson^a^			
Individual variables^b^	Pe_i_ (%)		aIRR	(95% CI)	PAF (%)	(95% CI)
Occupation^c^					**11.6**	**(8.4–14.6)**
Yes	57.65		1			
No	42.35		**1.38**	**(1.20–1.57)**		
Body mass index (kg/m^2^)					**5.2**	**(3.2–7.2)**
<25	57.84		1			
⩾25	42.16		**1.14**	**(1.00–1.30)**		
Knowledge score^d^					**7.3**	**(5.6–8.9)**
Two to four good answers	70.45		1			
Zero to one good answer	27.28		**1.23**	**(1.07–1.40)**		
Contextual variables^e^						
Dwelling					NA	
Collective	11.12		1			
Individual	88.88		1.00	(0.43–1.69)		
Chikungunya history in the neighbourhood^f^					NA	
No	6.49		1			
Yes or unspecified	93.53		1.00	(0.55–1.53)		
Chikungunya in the neighbours ^ dwelling					**45.9**	**(40.5–50.9)**
No	18.09		1			
Yes or unspecified and individual house	81.91		**2.28**	**(1.11–4.64)**		
Altitude of dwelling (m)					**13.1**	**(7.6–18.2)**
<250	72.72		**1.22**	**(1.05–1.41)**		
⩾250	27.28		1			
Socio-economic level of the municipality^g^					**19.0**	**(15.2–22.5)**
Advantaged	37.80		1			
Intermediate or deprived	62.20		**1.44**	**(1.25–1.65)**		
Maximal temperature at m-1 (°C)					**25.7**	**(21.4–29.8)**
Q1–Q2 ⩽ 28.522	36.44		1			
Q3–Q4 > 28.522	63.56		**1.68**	**(1.46–1.93)**		
Cumulated rainfall at m-1 (mm)					**12.6**	**(9.3–15.7)**
Q1–Q2 ⩽ 65	42.81		1			
Q3–Q4 > 65	57.19		**1.28**	**(1.12–1.46)**		
Overall PAF value	100		–		**80.8**	**(77.7–83.4)**

a Poisson regression model. Pe_i_: proportion of exposed individuals in the infected population are given as percentages. aIRR: adjusted incidence rate ratios and 95% CI: 95% confidence intervals. Population attributable fractions (PAF) and 95% CI are given as percentages.

b Individual variables are defined for personal (individual) exposures.

c Regular working or studying occupation.

d Score based on four questions (agree/disagree/1 point): Is chikungunya a mosquito-borne virus? Can chikungunya be transmitted by all species of mosquito? Can the mosquito transmit chikungunya to human? Can the human transmit the virus to the mosquito?

e Collective (grouping) variables are defined for contextual (household or area-level) exposures.

f Previous history of chikungunya fever in the neighbourhood indicative of clustering. Chikungunya in the neighbours ^ dwelling is an interaction term between the two abovementioned covariates.

g Derived from a homemade socio-economic index categorising the 24 municipalities of the island into tree levels based on three indices: socio-economic composition (three variables), spatial segregation of ethnic minorities (one variable) and existence of measures promoting social cohesion (one variable).

## Discussion

Here, we report the findings of a large serosurvey conducted on Reunion island, aimed to guide public health interventions for CHIKF control. By combining survey data and timely acquired climate variables, and using a multi-step multivariate analysis with Poisson regression models for dichotomous outcomes, our analysis has identified and weighed the community burden of individual and contextual determinants of CHIKF.

Interestingly, our results, which fulfil the standards of adequate public health impact measures for decision-making purposes, suggest a high disease burden of risk factors of primarily contextual origin, in comparison with that of individual determinants. In addition, we identified the most susceptible contextual variables to public health interventions.

Thus, in a nine-covariate Poisson model with an additional interaction term, the contributors for CHIKF, ranked by decreasing PAF values, were a CHIKF history in the neighbourhood and individual house, high maximal temperature the month preceding the infection, a socio-economically disadvantaged neighbourhood, low altitude of dwelling, high precipitations the month preceding the infection, occupational inactivity, poor knowledge on CHIKV transmission and obesity/overweight. Together, these covariates and their underlying causative factors uncovered over 80% of infections in the community.

Consistently, the addition of the maximal temperature and cumulated rainfall the month before the introduction of CHIKV in the area provided supportive information showing both an interaction and confounding between altitude and temperature. In accordance, the adjustment for climate variables decreased drastically the risk associated with altitude.

Of utmost importance, the overall PAF value of eight relevant contextual covariates and their underlying causative factors uncovered at least 89.5% of the CHIKF observed on the island, while the overall PAF value of eight relevant individual covariates and their underlying causative factors uncovered up to 44.1%.

This substantial role of contextual variables found in Reunion contrasts the results of the serosurvey conducted in Mayotte [14]. Herein, the overall PAF value of three independent household features (construction type, household size and Asset Index) was estimated to be 42% and combining the four independent individual characteristics (gender, birthplace, length of schooling, occupation) was estimated to be 89% in the same age population [14]. This discrepancy may underscore both the influence of different methods and distinctive contextual and individual characteristics of the disease burden. Even though epidemiologists have long recognised the ‘neighbourhood effects’ of social deprivation and climate as risk factors for vector-borne diseases such as dengue [24], health sociologists do emphasise the relevance of both categories of determinants, environmental changes and landscapes being primarily shaped by the habitat characteristics. Dwellings, in turn, harbour distinct populations with different mental (cognitive) representations and different behaviours against vector-borne diseases owed to different cultural or educational backgrounds [15].

The major burden of the altitude may underlie several explicative factors closely related, including climate variables, vegetation, host and dwelling densities. Herein, we show that the maximum risk was observed in the 250–500 m range, which coincides with the traditional Reunionese dwelling, the individual house with garden, an ideal niche for *Aedes* vectors breeding under tropical climate conditions [15], given a synergistic interaction between altitude and dwelling [25]. As expected, the effect of mid altitudes (250–750 m) was confounded by Tmax-1 and Pluv-1 in the nine-covariate final model, which unravels the critical roles of maximum temperature under 250 m and that of rainfall [25]. Importantly, ~80% of the Reunionese population lives under the height of 250 m along the coastal area of the island. *Aedes albopictus* was found as high as 1200 m in Reunion [26] and even as 2100 m in central Nepal [27]. Temperature and precipitations play a key role in CHIKV transmission [28]. Higher temperatures shorten the extrinsic incubation of CHIKV, lead to an increase in *Ae albopictus* vectors biting frequency and to an extension in their lifespan [29]. The effect of precipitations is dual [30]. Rainfalls create ideal conditions for mosquitoes to spawn [31], but may decrease host-seeking female abundance [32]. Drought periods often lead to inadequate water storage practices [33], whereas wet climates hasten the hatching of *Ae albopictus* eggs [27]. We believe these pathways likely underlie the effects of temperature and rainfall also in our context.

We found that a history of CHIKF in the neighbourhood, a proxy of disease clustering, uncovered a significant proportion of infections at population level. This is especially obvious, as spatiotemporal clustering is a feature of mosquito-borne diseases amenable to vector control interventions [27,30,31,34]. It has been well documented, both in urban and rural environments. This neighbourhood effect on CHIKV transmission dynamic was reported in three clusters investigated during the first epidemic wave in Reunion island [35], and is supported by mapping of participant's residencies revealing spatial heterogeneity of risk (data not shown). This influence of neighbourhood was later confirmed in Singapore [36]. It is also in agreement with more recent findings from a CHIKF outbreak in a small rural area of Bangladesh [37]. In this study, using Bayesian modelling of transmission, the authors reported that 58% of infections occurred at neighbourhood level.

The impact of individual house was found consistent with the findings of two other studies conducted on Reunion island [35,38]. Thus, in the three abovementioned clusters involved in the emergence, age, density and surface of the dwelling increased risk [35]. Moreover, makeshift constructions and the absence of window screens were associated with infection, consistent with other observations [14–16].

In our study, people living in socio-economically deprived areas were more likely to be infected, as previously shown for several infectious pathogens including CHIKV [38,39]. Indeed, neighbourhood environments (here assessed at municipality level) may contribute to CHIKV exposure due to residential segregation (i.e. disparities in dwelling places by race/ethnicity or socio-economic position) [40], or due to differences in mosquito control effectiveness, this latter being modified by household expenditures for chemically based protective measures, spatial coverage of interventions or community mobilisation (i.e. acceptability or reluctance behaviours) [41–43]. This theoretical framework was confirmed in Mayotte where social disparities in infection rate were primarily structured by housing conditions as well as cognitive representations of the disease, ‘legitimate images’ being found in rich urban settings, ‘folk theories’ being found in poor suburban settings [15]. Interestingly, the neighbourhood environment was found different between dengue (or Zika) and CHIKF-affected populations in French Guiana and Rio Janeiro, Brazil, where CHIKF tended to impact the most impoverished communities living in overcrowded areas, which may explain the higher basic reproductive number (R0) for CHIKV than for flaviviruses [44,45].

With respect to individual variables, occupational inactivity was a key determinant of infection, which is likely coherent with increasing exposure to mosquito bites in the home outdoor environment. In agreement, outdoor activities (e.g. being employed as farm worker) have been increasingly reported as a risk factor for CHIKF [17]. In our study, farm workers were the most at-risk profession. We believe that the effect of occupational inactivity may thus reflect the seasonal and undeclared character of farm work on Reunion island, or the importance of other on-off outdoor jobs in a community largely affected by unemployment.

We found knowledge of the disease having an impact on infection. Knowledge of a disease is believed to drive attitudes, beliefs and practices towards better protection [46]. Interestingly, appropriate behaviours were not shown to be protective when knowledge was adjusted. Poor knowledge on CHIKV transmission was relevant among elder individuals (⩾80 years), indigenous, illiterate or living alone, which sheds light on the vulnerability of Creole populations. These data are coherent with the irrational ideas of catastrophe thinking reported as ‘folk theories’ among Indian ocean communities along CHIKF epidemics, where the absence of perceived controllability was the most common belief [15,38]. They are also in line with the findings of the KAP study in French Guiana, which revealed that adoption of protective behaviours against vector-borne diseases is a multi-factorial process that depends on both socio-economic and cognitive factors [46].

Last, obesity and to a lesser extent being overweight were slightly associated with infection, which may likely be the conjunction of several possible factors: lack of education and illiteracy, lesser physical activity, larger body surface, higher *Aedes* mosquito response to CO_2_, lactic acid or sweat compounds [47].

Our study has some strengths and limitations. First, it was population-based, and our estimates were weighted on the sampling fraction to ensure the best possible representativeness. Notwithstanding, our sample was a little shifted towards women and elderly people and we had to correct the weighting of the sample upon analysis to minimise the selection bias. Second, we used a Poisson regression model for dichotomous outcomes to overcome convergence problems. In the framework of cross-sectional studies, this user-friendly model has gained credit over the last decade to be increasingly used as a good proxy of the log binomial model, including in seroepidemiologic studies [18,21]. In our study, the necessity to compensate the selection bias using survey-readjusted estimates (reweighted on the sampling plan) precluded us to use with Stata the robust variance option, conditional to this model [21]. However, the estimates of our final model did not differ from unweighted estimates with robust variance (data not shown), so that it is unlikely that this statistical constraint might have changed the overall sense of our results. Third, the use of a Kish sampling method did not allow assessing intra-household random effects, which likely biased the neighbourhood effect estimates. Given the clustering of CHIKF cases [38], we strongly recommend cluster sampling for investigating future outbreaks [14]. Fourth, the KAP component of the survey was likely sensitive to social desirability bias [48], which may have limited the interpretation of the role of protective behaviours. This was suggested in our study by the absence of positive correlation between the knowledge score and the behaviour score. Alternatively, identification of clusters may have also promoted protective behaviours within the infested areas, which may have skewed the relationship between protective behaviours and infections towards a negative correlation.

In conclusion, our findings lend support to a major role of contextual risk factors in CHIKF outbreaks, which in turn highlights the appropriateness of community-based interventions in socio-economically disadvantaged and vector-friendly neighbourhoods [49]. Investigating pathways linked to socio-environmental determinants might thus be useful to unravel CHIKF causative factors and to understanding the drivers of future arboviral emergences.

## Supplementary material

For supplementary material accompanying this paper visit http://doi.org/10.1017/S0950268818000341. click here to view supplementary material
