# Supplementary material for: Individual and contextual risk factors for chikungunya virus infection: the SEROCHIK cross-sectional population-based study
Source: Epidemiol Infect. 2018 May 3;146(8):1056–64. doi: 10.1017/S0950268818000341 (PMC5998769; doi:10.1017/S0950268818000341)
Supplement: Supplementary file 1 [file S0950268818000341sup001.docx]

**Supplementary material (online publication only)**

**Supplemental file 1**

| **Table S1.** **Bivariate analysis risk estimates of individual risk factors for chikungunya among 2,101 subjects (≥ 15 years), SEROCHIK survey, August-October 2006, Reunion island** | | | | | | |
| --- | --- | --- | --- | --- | --- | --- |
| **Bivariate model** | |  | | | **Poisson ^(1)^** | |
| **Individual variables^†^** | **dbSP (%)** | | **(95% CI)** | **cIRR** | | **(95% CI)** |
| **Gender** |  | |  |  | |  |
| Women | 40.38 | | (37.59, 43.16) | 1 | |  |
| Men | 41.31 | | (38.10, 44.52) | 1.02 | | (0.90, 1.16) |
| **Age** |  | |  |  | |  |
| 15 to 29 years | 37.67 | | (33.35, 41.98) | 1 | |  |
| 30 to 39 years | 36.65 | | (31.77, 41.52) | 0.97 | | (0.79, 1.19) |
| 40 to 49 years | 39.29 | | (34.49, 44.09) | 1.04 | | (0.86, 1.26) |
| 50 to 59 years | 44.06 | | (38.90, 49.22) | 1.17 | | (0.96, 1.42) |
| 60 to 69 years | 50.16 | | (43.88, 56.43) | **1.33** | | **(1.09, 1.61)** |
| ≥70 years | 56.02 | | (49.77, 62.26) | **1.49** | | **(1.23, 1.80)** |
| **Chronic disease ^¶^** |  | |  |  | |  |
| No | 37.63 | | (34.99, 40.26) | 1 | |  |
| Yes | 48.28 | | (44.72, 51.83) | **1.28** | | **(1.13, 1.44)** |
| Unspecified | 26.45 | | (3.34, 49.55) | 0.70 | | (0.29, 1.69) |
| **Occupation*** |  | |  |  | |  |
| Yes | 32.94 | | (29.92, 35.96) | 1 | |  |
| No | 48.81 | | (45.80, 51.81) | **1.48** | | **(1.30, 1.69)** |
| **Body mass index** |  | |  |  | |  |
| < 25 kg/m^2^ | 37.27 | | (34.60, 39.94) | 1 | |  |
| 25 to 29.9 kg/m^2^ | 43.92 | | (39.96, 47.88) | **1.16** | | **(1.01, 1.34)** |
| ≥30 kg/m^2^ | 54.62 | | (47.44, 61.79) | **1.45** | | **(1.21, 1.74)** |
| **Knowledge score**** |  | |  |  | |  |
| Four good answers | 32.94 | | (28.54, 37.33) | 1 | |  |
| Three good answers | 38.01 | | (34.12, 41.90) | 1.15 | | (0.94, 1.40) |
| Two good answers | 39.53 | | (34.78, 44.27) | 1.20 | | (0.97, 1.48) |
| One good answer | 49.26 | | (43.26, 55.26) | **1.50** | | **(1.20, 1.85)** |
| Zero good answer | 55.85 | | (49.37, 62.32) | **1.70** | | **(1.37, 2.09)** |
| **Self-protection with repellent sprays/creams** |  | |  |  | |  |
| Yes | 37.01 | | (35.06, 38.96) | **0.82** | | **(0.72, 0.93)** |
| No | 44.94 | | (42.93, 46.94) | 1 | |  |
| **Self-protection with insecticides spreading^#^** |  | |  |  | |  |
| Yes | 37.51 | | (35.08, 39.22) | **0.88** | | **(0.77, 0.99)** |
| No | 42.79 | | (40.67, 44.91) | 1 | |  |
| **Behaviour score^# #^** |  | |  |  | |  |
| 0 | 36.69 | | (34.67, 38.71) | 1 | |  |
| 1 to 3 | 36.79 | | (34.77, 38.80) | 1.00 | | (0.81, 1.24) |
| 4 to 5 | 40.47 | | (38.41, 42.52) | 1.10 | | (0.88, 1.37) |
| 6 to 11 | 49.61 | | (47.51, 51.70) | **1.35** | | **(1.09, 1.68)** |
| ^(1)^ Poisson regression model; dbSP: design-based seroprevalence; cIRR: crude incidence rate ratios.  **^†^** Individual variables are defined for personal (individual) exposures. **^¶^** Hypertension, diabetes mellitus, hypercholesterolemia, chronic heart disease, asthma, rheumatism; * employment or ongoing studies/inactivity; ** score based on four questions (agree/disagree/ 1 point): Is Chikungunya a mosquito-borne virus? Can Chikungunya be transmitted by all species of mosquito? Can the mosquito transmit Chikungunya to human? Can the human transmit the virus to the mosquito? ; **^#^** self-use of home indoor spreading; **^# #^** score based on eleven paradoxical behaviours (yes/no, each 1 point): covering tanks and water supplies, putting sand in containers, pruning shrubs and cleaning wastes, removing clutter in the courtyard, removing sub-pots, emptying containers, emptying pools and basins, checking drainage of gutters, cleaning the garden or the balcony, removing the trees and plant leaves, and removing rubbish. | | | | | | |

**Supplemental file 2**

| **Table S2. Factors linked to poor knowledge on chikungunya transmission (score < 2 points) among 2,101 subjects (≥ 15 years), SEROCHIK survey, August-October 2006, Reunion island** | | | | |
| --- | --- | --- | --- | --- |
| **Variable** | **Score < 2 points (%)** | **cIRR** | **(95 % CI)** | ***P* value** |
| Gender male  female | 48.0  48.3 | 0.9  1 | (0.7, 1.1) | 0.339 |
| Age15 – 19 years20 – 29 years30 – 39 years40 – 49 years50 – 59 years60 – 69 years70 – 79 years ≥ 80 years | 41.6  43.7  43.4  49.4  48.6  60.5  69.6  82.8 | 0.6  1  1.0  1.3  1.1  1.2  1.3  2.5 | (0.4, 1.0)  (0.7, 1.5)  (0.9, 1.9)  (0.7, 1.6)  (0.8, 2.0)  (0.7, 2.3)  (1.2, 5.6) | 0.076 |
| Place of birthLa Réunion Other Indian Ocean Islands  Mainland France  Others | 51.5  46.3  24.9  19.4 | 1  **0.8**  **0.4**  **0.3** | **(0.4, 1.3)**  **(0.3, 0.6)**  **(0.1, 0.8)** | **< 0.001** |
| Marital statusCelibacy Cohabitation  Marriage  Separation/widowhood  Divorce | 47.1  43.4  46.7  74.5  43.9 | **1.3**  1.0  1  **1.8**  0.9 | **(1.0, 1.8)**  (0.7, 1.4)  1  (**1.2, 2.9)**  (0.6, 1.5) | **0.025** |
| Literacy (reading skills)No Yes | 58.1  21.6 | **5.4**  1 | **(2.6, 5.3)** | **< 0.001** |
| cIRR: crude incidence rate ratios. * Knowledge score based on four questions (agree/disagree/ 1 point): Is Chikungunya a mosquito-borne virus? Can Chikungunya be transmitted by all species of mosquito? Can the mosquito transmit Chikungunya to human? Can the human transmit the virus to the mosquito? | | | | |

**Supplemental file 3**

| **Table S3.** **Multivariate full-models estimates of individual risk factors for chikungunya among 2,101 subjects (≥ 15 years), SEROCHIK survey, August-October 2006, Reunion island** | | |
| --- | --- | --- |
| **Multivariate model** | **Poisson ^(1)^** | |
| **Individual variables^†^** | **aIRR** | **(95% CI)** |
| **Gender** |  |  |
| Women | 1 |  |
| Men | 1.07 | (0.93, 1.23) |
| **Age** |  |  |
| 15 to 29 years | 1 |  |
| 30 to 39 years | 0.93 | (0.75, 1.15) |
| 40 to 49 years | 0.94 | (0.75, 1.16) |
| 50 to 59 years | 1.00 | (0.80, 1.23) |
| 60 to 69 years | 0.98 | (0.77, 1.24) |
| ≥70 years | 1.09 | (0.86, 1.37) |
| **Chronic disease^¶^** |  |  |
| No | 1 |  |
| Yes  Unspecified | 1.11  0.47 | (0.94, 1.30)  (0.14 – 1.55) |
| **Occupation*** |  |  |
| Yes | 1 |  |
| No | **1.42** | **(1.20, 1.66)** |
| **Body mass index** |  |  |
| < 25 kg/m^2^ | 1 |  |
| 25 to 29.9 kg/m^2^ | 1.09 | (0.93, 1.27) |
| ≥30 kg/m^2^ | **1.29** | **(1.05, 1.58)** |
| **Self-protection with repellent sprays/creams** |  |  |
| Yes | 1.08 | (0.94 – 1.25) |
| No | 1 |  |
| **Knowledge score**** |  |  |
| Four good answers | 1 |  |
| Three good answers | 1.12 | (0.91, 1.37) |
| Two good answers | 1.11 | (0.89, 1.39) |
| One good answer | **1.27** | **(1.01, 1.59)** |
| Zero good answer | **1.33** | **(1.05, 1.69)** |
| **Self-protection with insecticides spreading^#^** |  |  |
| Yes | 1.09 | (0.94 – 1.26) |
| No | 1 |  |
| **Population attributable fraction**  **associated with the model (%)** | **PAF** | (**95% CI**) |
|  | 44.1% | (41.5%, 46.6%) |
| ^(1)^ Poisson regression model; aIRR: adjusted incidence rate ratios.  **^†^** Individual variables are defined for personal (individual) exposures. **^¶^** Hypertension, diabetes mellitus, hypercholesterolemia, chronic heart disease, asthma, rheumatism; * employment or ongoing studies/inactivity; ** score based on four questions (agree/disagree/ 1 point): Is Chikungunya a mosquito-borne virus? Can Chikungunya be transmitted by all species of mosquito? Can the mosquito transmit Chikungunya to human? Can the human transmit the virus to the mosquito ?; **^#^** self-use of home indoor spreading. | | |

**Supplemental file 4**

| **Table S4.** **Bivariate analysis risk estimates of contextual risk factors for chikungunya among 2,101 subjects (≥ 15 years), SEROCHIK survey, August-October 2006, Reunion island** | | | | | | | |
| --- | --- | --- | --- | --- | --- | --- | --- |
| **Bivariate model** | | |  | | | **Poisson ^(1)^** | |
| **Contextual variables^‡^** | | **dbSP (%)** | | **(95% CI)** | **cIRR** | | **(95% CI)** |
| **Dwelling** | |  | |  |  | |  |
| Collective | | 24.58 | | (20.42, 28.74) | 1 | |  |
| Individual | | 46.25 | | (43.72, 48.77) | **1.88** | | **(1.52, 2.32)** |
| **Household size** | |  | |  |  | |  |
| 1 inhabitant | | 42.60 | | (38.26, 46.94) | 1.12 | | (0.98, 1.27) |
| 2 to 4 inhabitants | | 38.01 | | (35.37, 40.65) | 1 | |  |
| ≥ 5 inhabitants | | 46.03 | | (40.41, 51.64) | **1.21** | | **(1.04, 1.40)** |
| **Chikungunya history in the neighbourhood^#^** | |  | |  |  | |  |
| No | | 20.57 | | (15.70, 25.44) | 1 | |  |
| Yes  Unspecified | | 48.06  32.02 | | (45.25, 50.87)  (28.20, 35.84) | **2.33**  **1.56** | | **(1.76, 3.09)**  **(1.14, 2.11)** |
| **Altitude of dwelling** | |  | |  |  | |  |
| < 250 meters | | 43.06 | | (40.50, 45.61) | **2.74** | | **(1.60, 4.68)** |
| 250 to 499 meters | | 46.47 | | (40.85, 52.09) | **2.95** | | **(1.70, 5.13)** |
| 500 to 749 meters | | 38.36 | | (30.72, 45.99) | **2.44** | | **(1.36, 4.36)** |
| 750 to 999 meters | | 19.63 | | (12.13, 27.12) | 1.25 | | (0.63, 2.46) |
| ≥1000 meters | | 15.74 | | (8.08, 23.39) | 1 | |  |
| **Residential area** | |  | |  |  | |  |
| North | | 30.43 | | (26.39, 34.47) | 1 | |  |
| West | | 44.48 | | (40.20, 48.75) | **1.46** | | **(1.19, 1.79)** |
| South | | 40.75 | | (37.32, 44.17) | **1.34** | | **(1.10, 1.63)** |
| East | | 52.97 | | (47.24, 58.69) | **1.74** | | **(1.41, 2.15)** |
| **Socio-economic level of**  **the municipality^##^** | |  | |  |  | |  |
| Advantaged | | 32.10 | | (29.22, 34.97) | 1 | |  |
| Intermediate | | 46.73 | | (42.22, 51.24) | **1.46** | | **(1.24, 1.71)** |
| Deprived | | 50.30 | | (46.36, 54.24) | **1.57** | | **(1.36, 1.81)** |
| ^(1)^ Poisson regression; dbSP: design-based seroprevalence; cIRR: crude incidence rate ratios.  **^‡^** Collective (grouping) variables are defined for contextual (household or area-level) exposures.  **^#^** Previous history of chikungunya fever in the neighbourhood indicative of clustering; **^##^** Derived from a homemade socio-economic index categorising the 24 municipalities of the island into tree levels based on three indices: socio-economic composition (3 variables), spatial segregation of minorities (1 variable), existence of measures promoting social cohesion (1 variable). | | | | | | | |

**Supplemental file 5**

| **Table S5. Effect of climate variables at month-1, altitude and residential area on chikungunya among 2,379 subjects, SEROCHIK survey, August-October 2006, Reunion island** | | | | |
| --- | --- | --- | --- | --- |
| **Multivariate final model** | | **Poisson ^(1)^** | | |
| **Climate variables** (continuous) | | **aIRR** | **(95% CI)** | ***P value*** |
| **Mean solar radiation at m-1** | | 1.000 | (0.999 – 1.000) | 0.211 |
| **Maximal temperature at m-1** | | **1.14** | **(1.104 – 1.187)** | **< 0.001** |
| **Cumulated Rainfall at m-1** | | **1.001** | **(1.000 – 1.002)** | **< 0.001** |
| **Geographic cluster variables** | |  |  |  |
| **Altitude** (continuous) | | **0.999** | **(0.999, 1.000)** | **0.043** |
| **Residential area** | |  |  |  |
| North | | 1 |  |  |
| West | | 1.144 | (0.919, 1.425) | 0.228 |
| South | | **1.330** | **(1.096, 1.613)** | **0.004** |
| East | | **1.452** | **(1.185, 1.779)** | **< 0.001** |
| This model was developed in the whole population. ^(1)^ Poisson regression model without robust variance option; aIRR: adjusted incidence rate ratios (risk ratio). Other climate variables were dropped out from the model for non-significance or autocorrelation (multicolinearity): mean solar radiation at m0, maximal temperature at m0, minimal temperature at m-1, minimal temperature at m0, average temperature at m-1, average temperature at m0, cumulated rainfall at m-2, cumulated rainfall at m-1. | | | |  |

**Supplemental file 6**

**Figure S6. Design-based estimates for the seroprevalence of chikungunya, taken as a smooth functions of maximal temperature and cumulated rainfall at the month preceding the introduction of CHIKV, SEROCHIK survey, August-October 2006, Reunion island.**


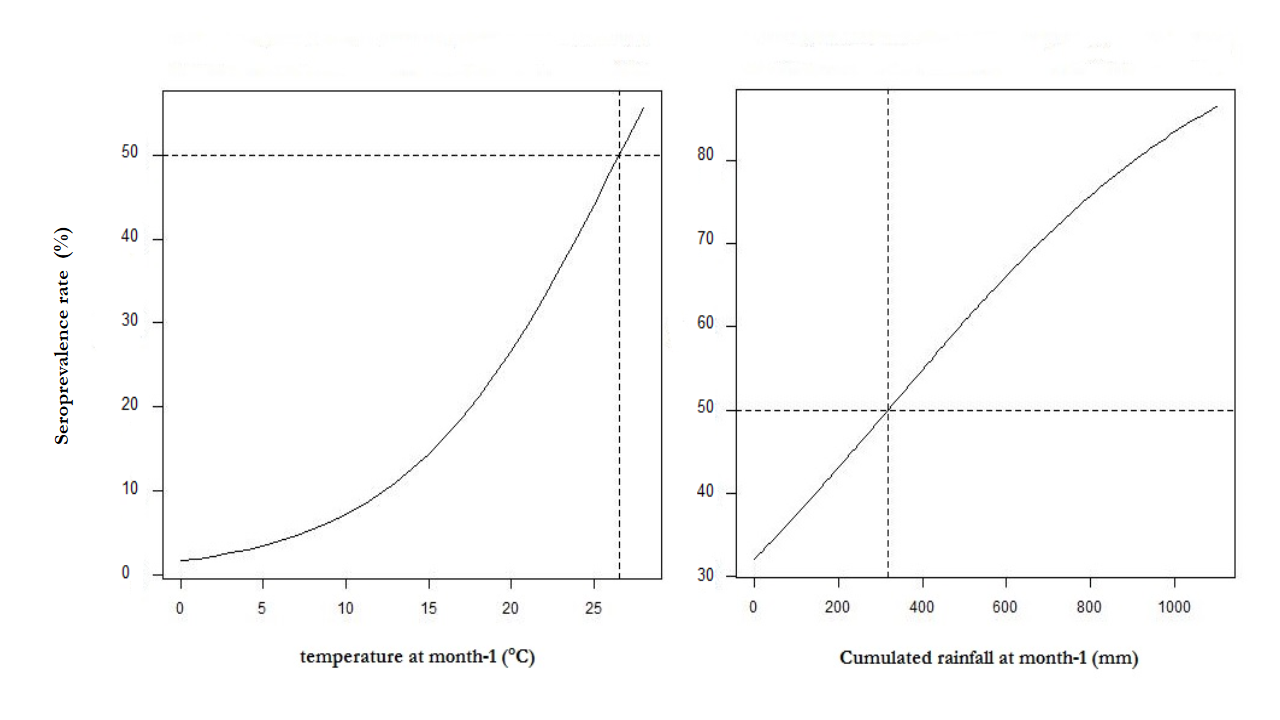


**Supplemental file 7**

| **Table S7.** **Multivariate full-model estimates of contextual risk factors for chikungunya among 2,101 subjects (≥ 15 years), SEROCHIK survey, August-October 2006, Reunion island** | | |
| --- | --- | --- |
| **Multivariate model** | **Poisson ^(1)^** | |
| **Contextual variables** | **aIRR** | **(95% CI)** |
| **Dwelling** |  |  |
| Collective | 1 |  |
| Individual | **1.67** | **(1.34, 2.08)** |
| **Household size** |  |  |
| 1 inhabitant | **1.15** | **(1.01, 1.30)** |
| 2 to 4 inhabitants | 1 |  |
| ≥ 5 inhabitants | 1.08 | (0.93, 1.24) |
| **Chikungunya history in the neighbourhood^#^** |  |  |
| No | 1 |  |
| Yes  Unspecified | **1.92**  **1.49** | **(1.41, 2.60)**  **(1.07, 2.07)** |
| **Altitude of dwelling** |  |  |
| < 250 meters | **1.94** | **(1.10, 3.43)** |
| 250 to 499 meters | 1.74 | (0.94, 3.21) |
| 500 to 749 meters | 1.85 | (0.99, 3.44) |
| 750 to 999 meters | 1.22 | (0.60, 2.47) |
| ≥1000 meters | 1 |  |
| **Residential area** |  |  |
| North | 1 |  |
| West | 1.06 | (0.85, 1.33) |
| South | 1.08 | (0.86, 1.34) |
| East | 1.03 | (0.80, 1.31) |
| **Socio-economic level of the municipality^##^** |  |  |
| Advantaged | 1 |  |
| Intermediate | **1.31** | **(1.05, 1.64)** |
| Deprived | **1.33** | **(1.12, 1.56)** |
| **Maximal temperature at m-1** |  |  |
| Q1-Q2 ≤ 28.522 °C | 1 |  |
| Q3-Q4 > 28.522 °C | **1.59** | **(1.37, 1.83)** |
| **Cumulated rainfall at m-1** |  |  |
| Q1-Q2 ≤ 65 mm | 1 |  |
| Q3-Q4 > 65 mm | **1.31** | **(1.15 – 1.49)** |
| **Population attributable fraction**  **associated with the model (%)** | **PAF** | (**95% CI**) |
|  | 89.5% | (83.4%, 93.3%) |
| ^(1)^ Poisson regression model. aIRR: adjusted incidence rate ratios.  **^‡^** Collective (grouping) variables are defined for contextual (household or area-level) exposures. **^#^** Previous history of chikungunya fever in the neighbourhood indicative of clustering; **^##^** Derived from a homemade socio-economic index categorising the 24 municipalities of the island into tree levels based on three indices: socio-economic composition (3 variables), spatial segregation of ethnic minorities (1 variable), existence of measures promoting social cohesion (1 variable). | | |

**Supplemental file 8**

**Appendix 1. Description of the study setting.**

Reunion island is a French overseas territory of 787,836 inhabitants, located in the *Mascarene* archipelago, south-western part of the Indian Ocean (21° S latitude). Its tropical climate can be summarized in two seasons: a warm rainy season (November to April), propitious to the multiplication of vectors, and a temperate dry season (May to October) swept by trade winds. Its settlement, begun in the 17^th^ century, led to a massive deforestation along the coasts, where the primary tropical forest was replaced by sugarcane monoculture, and urbanization. Since departmentalization in 1946, standards of living and health care have improved for the community which strengthened paradoxically social disparities in access to care [i]. These socio-economic changes supported the development of trade and tourism with other neighbouring islands, which exposed Reunion to the introduction of vectors and arboviruses *via* air or sea transport [ii]. In 1978, the island faced a massive Dengue (DEN-2) virus outbreak with a putative attack rate of 30-35% [iii].

**Additional references**

1. **Vaillant A.** La Réunion, un cloisonnement social et spatial. In: La Réunion, koman i lé ? Territoire, Santé, Société. Ed: Presses Universitaires de France, Paris, France, 2008; pp 17-50.
2. **Tortosa P, *et al***. Deciphering arboviral emergence within insular ecosystems. *Infection and Genetic Evolution* 2012; **12**: 1333-1339.
3. **Michault A.** Insularité et risque épidémique. *Bulletin de la Société de Pathologie Exotique* 1998; **91**: 52-55.

**Supplemental file 9.**

**Appendix 2.** **Questionnaires of the SEROCHIK seroprevalence study**


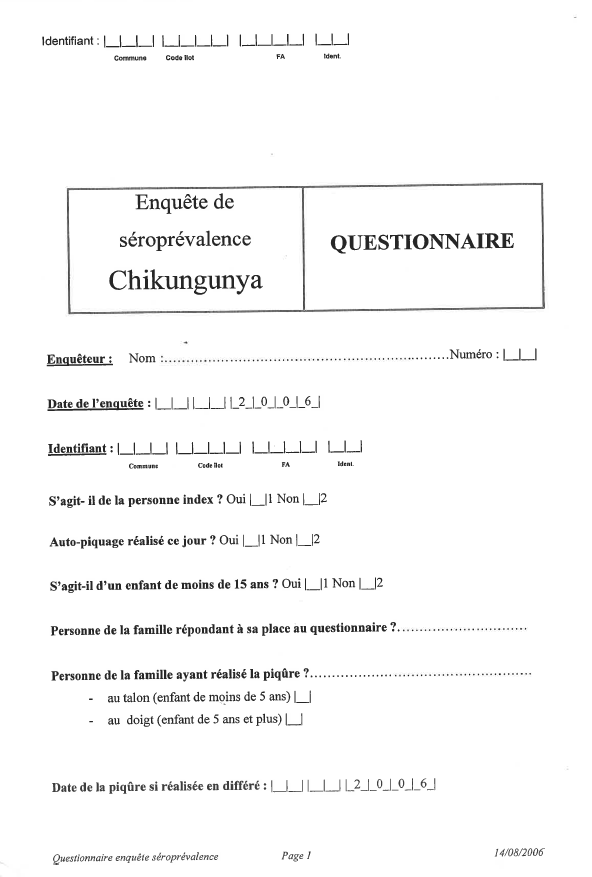


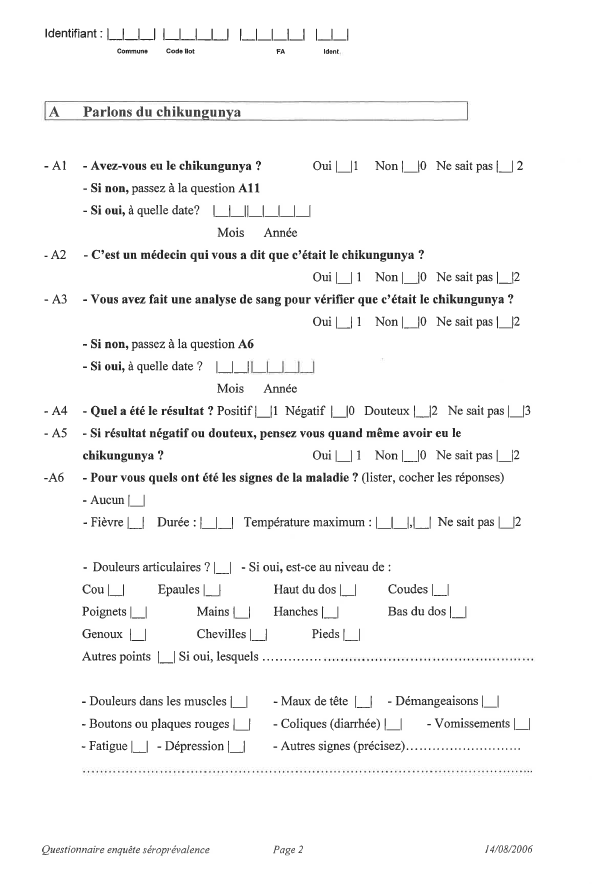


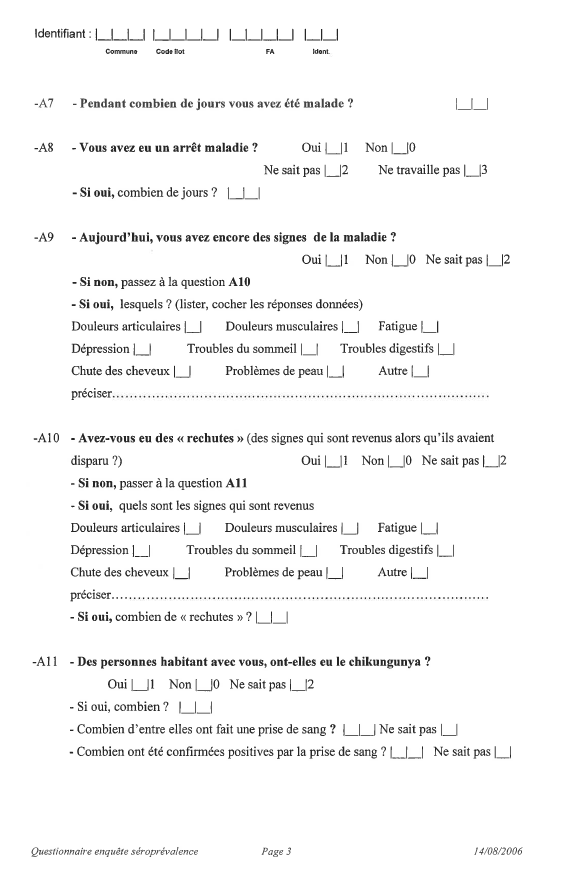


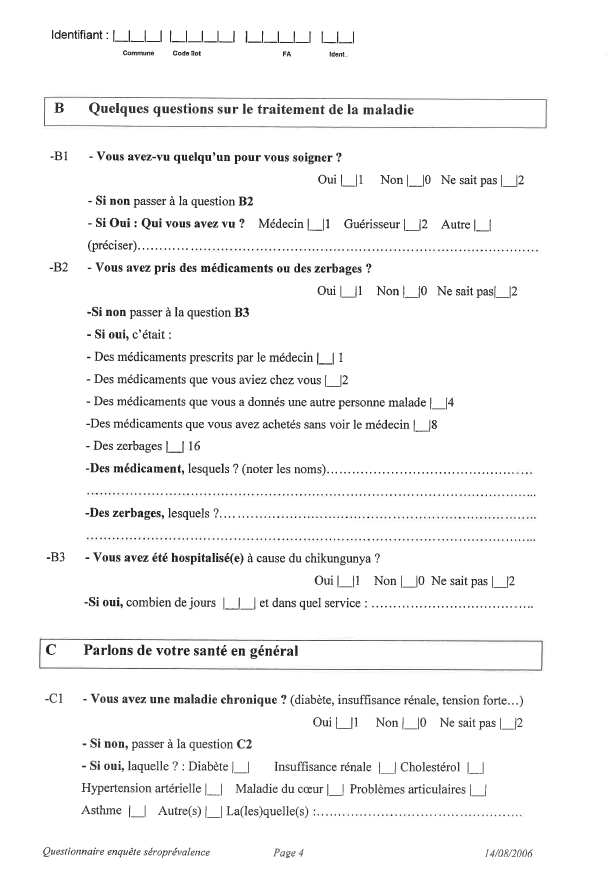


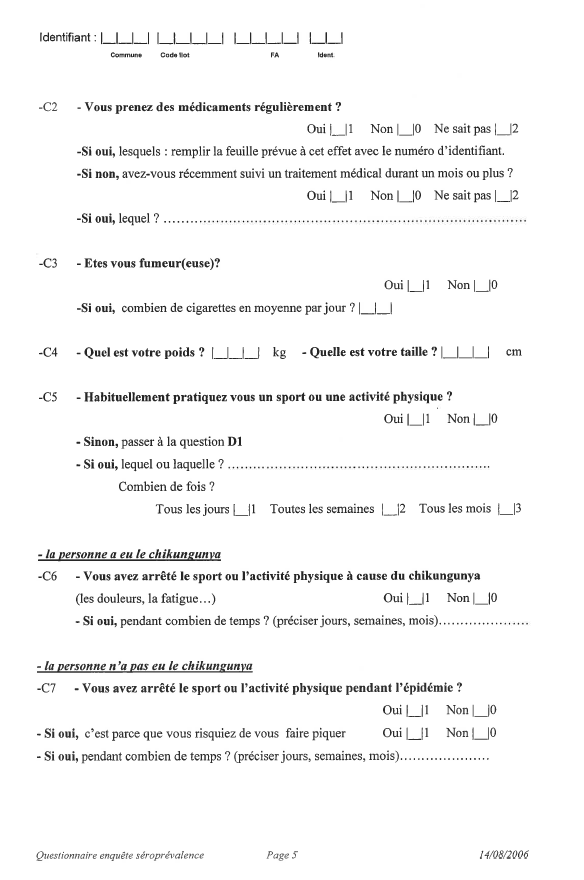


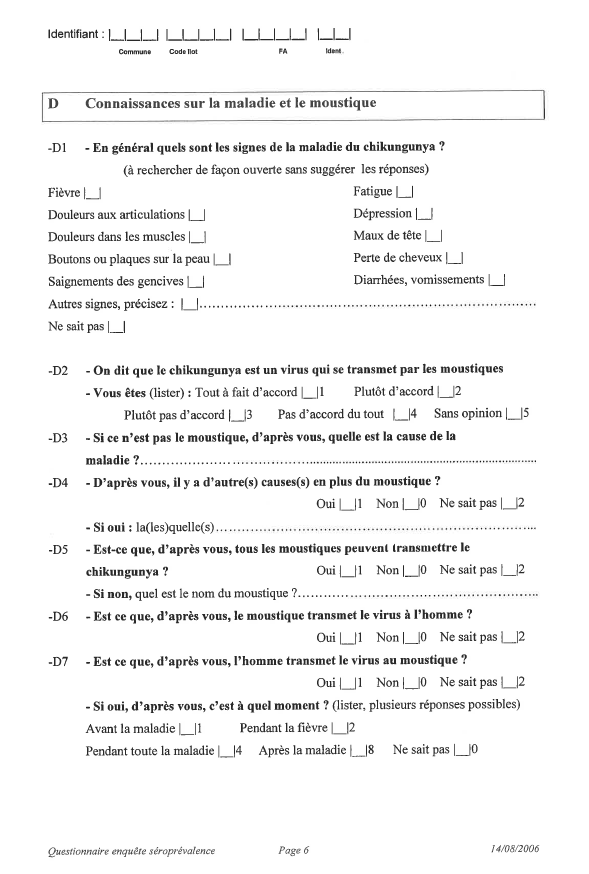


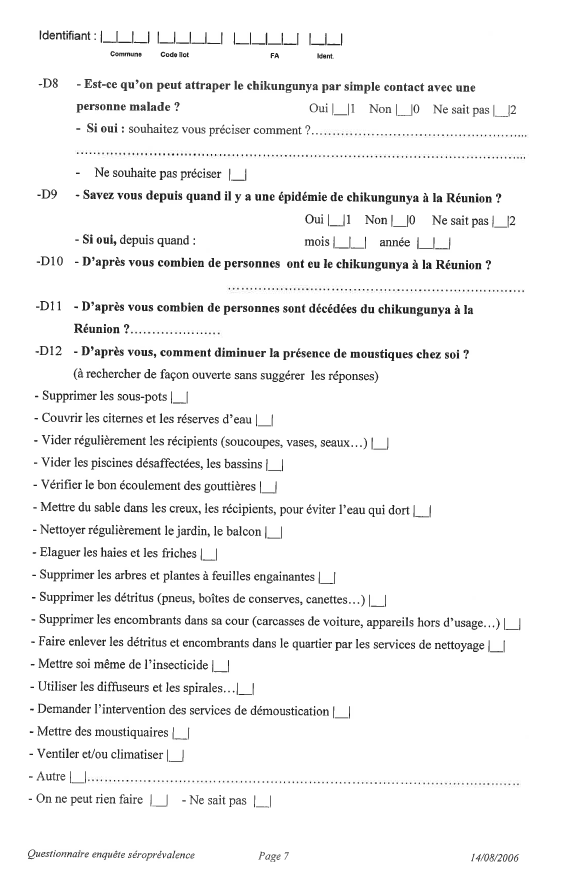


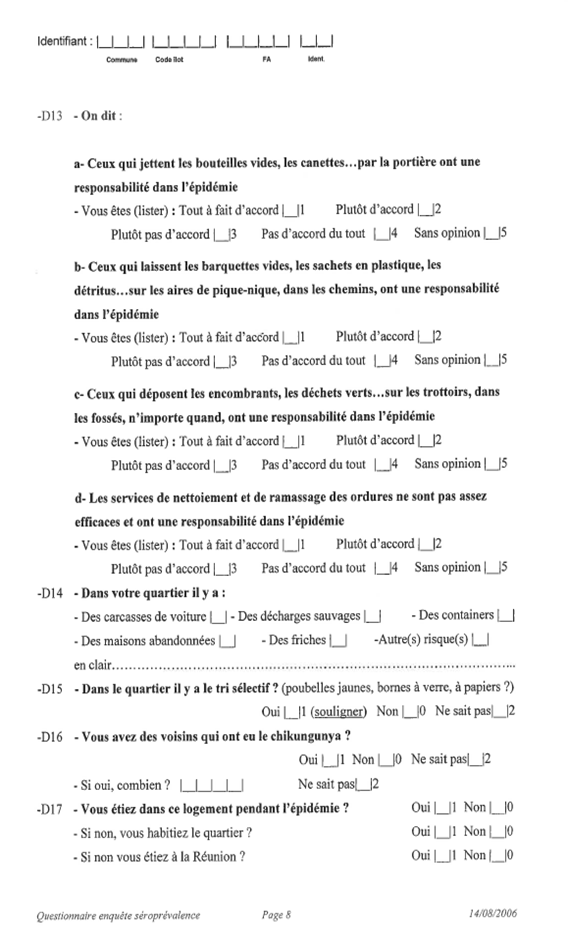


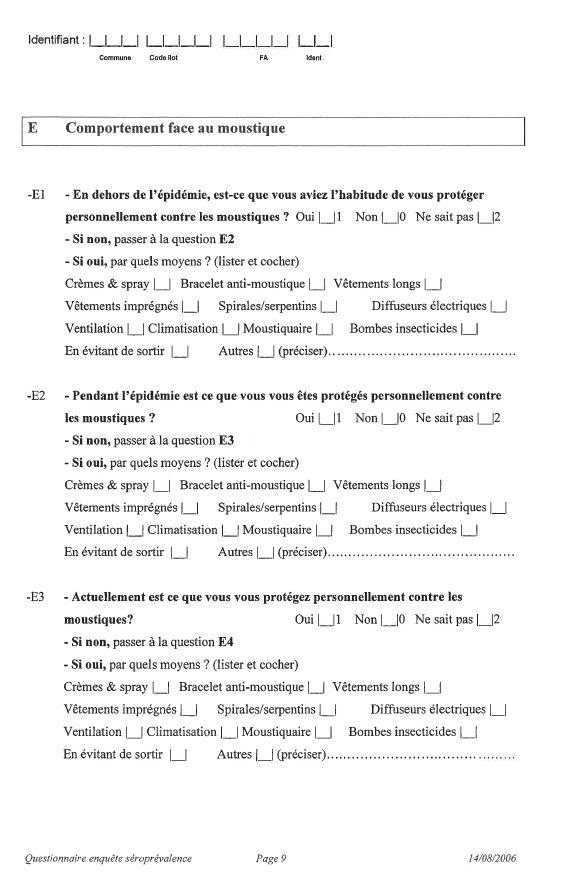


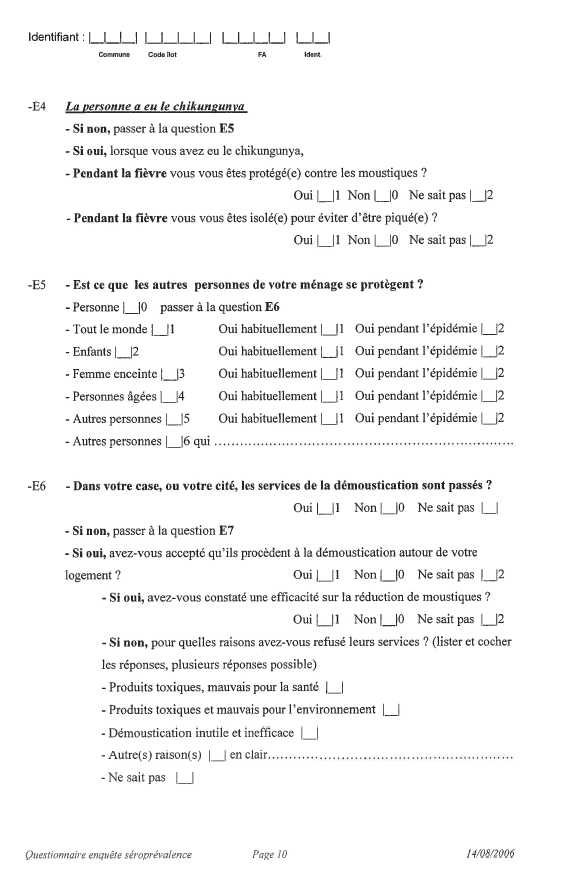


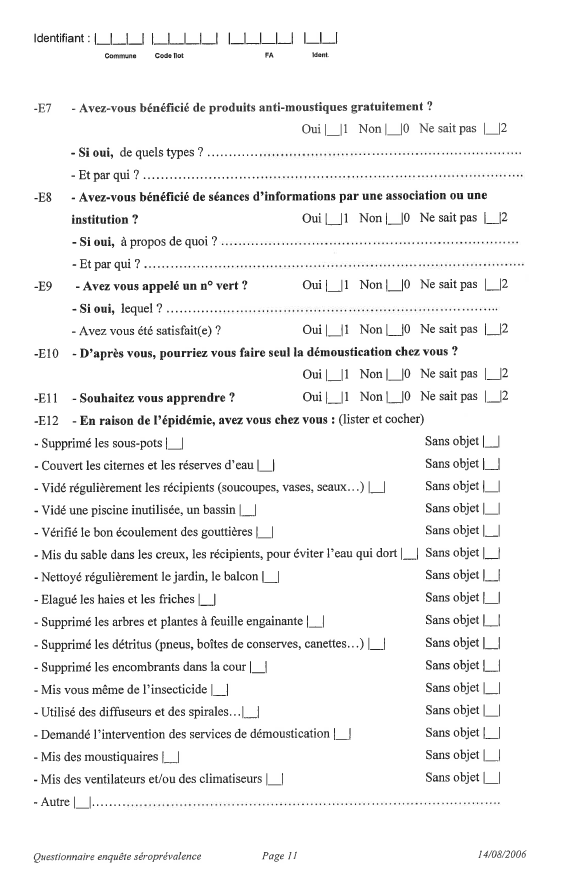


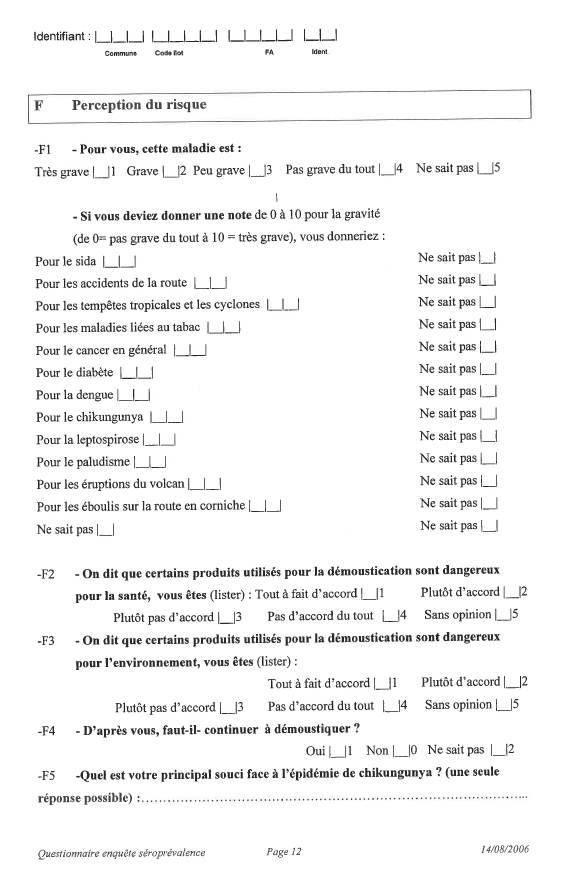


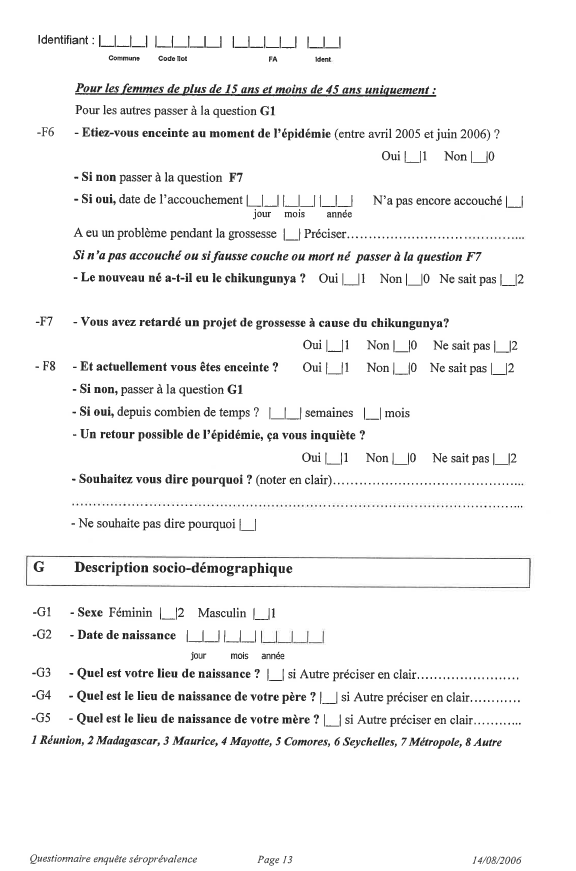


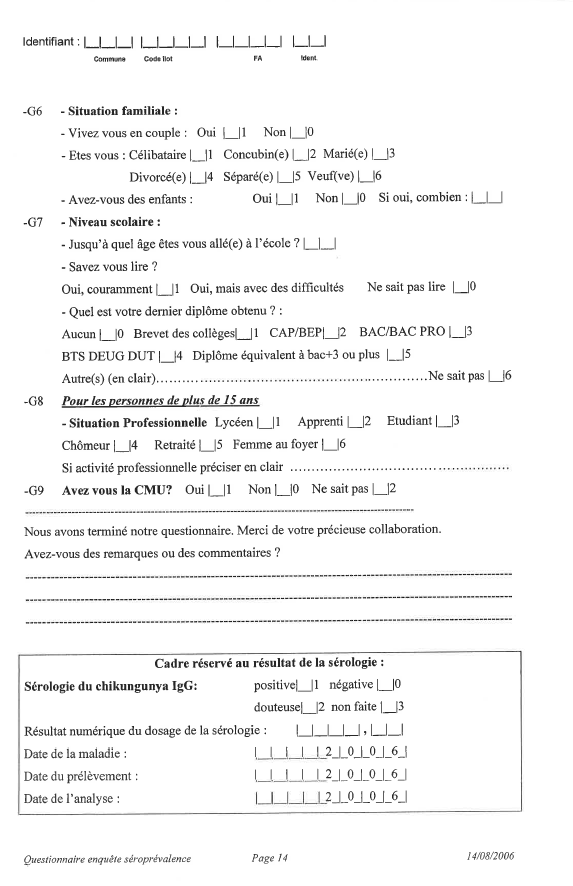


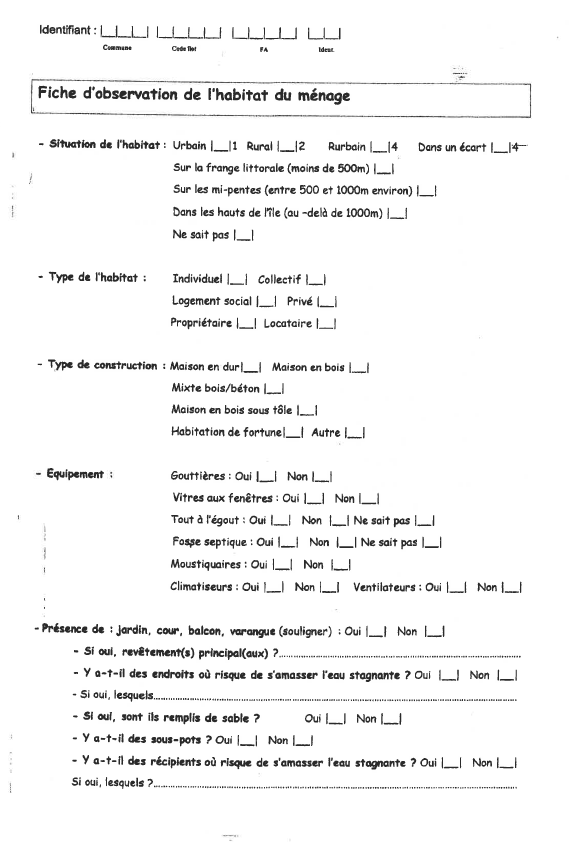


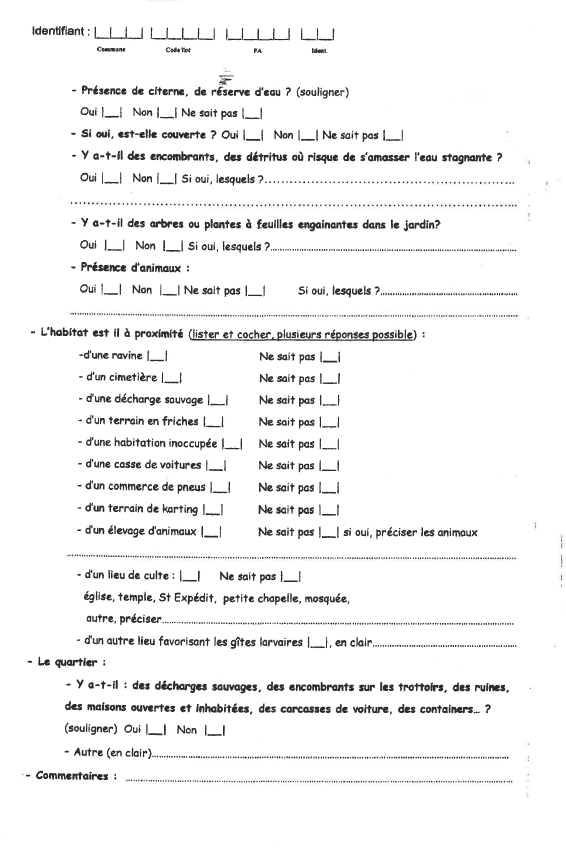


**Supplemental file 10**

**Appendix 3. Social deprivation index of the socio-economic level of the municipality.**

Briefly, it was structured on three scales gathering classical components of social epidemiology for characterizing area-level data [iv]. These latter were chosen both from freely available census-based information and from the data of a birth registry

The first scale was a deprivation index characterizing the socio-economic composition of the municipalities, which combined the average tax income per household, the number of taxable tax households, and the proportion of individuals earning the minimum income. Each component was scored 0 to +2 points, with respect to the terciles of each variable. Its average score ranged 0 (minimum deprivation) to +2 points (maximum deprivation).

The second scale measured the social structure of the municipalities based on the segregation of minorities. Briefly, the place of birth, a proxy of the ethnic group on Reunion island was categorised for each municipality of residency into seven classes according the proportions of minorities (native from Mayotte, Madagascar, Comoro islands, mainland France, other) and compared to regional averages (native from Mayotte and Comoro being considered as deprived). With respect to the overrepresentation or underrepresentation of minorities, the municipality was classified between -2 (maximum wealth) and +2 (maximum deprivation).

The third scale was an indicator of social cohesion based on the presence or absence of CUCS (urban social cohesion contract) in the municipality [v]. CUCS is a contract between the French state and local city governments that commits both partners to implement joint actions aimed at improving the daily life of residents in neighbourhoods experiencing difficulties (unemployment, violence, difficulties in housing). Its average score ranged -1 (minimum deprivation) to +1 point (maximum deprivation).

These three scales were summed to provide an overall social deprivation index with three categories: -3 to 0 (advantaged), 1 to +2 (intermediate), +3 to +5 (deprived).

**Additional references**

1. **Kaufman JS.** Social epidemiology. In Modern Epidemiology. 3^rd^ edition. Ed:

Lippincott Williams & Wilkins, Philadelphia, USA, 2008; 532-548 pp.

1. **Ministère de la Ville, de la Jeunesse et des Sports**, 2011. https// : sig.ville.gouv.fr/page/45.

**Supplemental file 11**

**Appendix 4. Full technique of blood testing.**

Capillary blood was collected from 2442 participants by finger-prick using a sterile, single use lancet and absorbed on filter paper (Whatman 903® Protein Saver™ Card, Schleicher & Schuell) [vi]. Filter papers were allowed to air dry, placed in individual, labelled envelopes and stored at 4 °C. Antibodies were eluted from a c. 6 mm blood stain section, which was cut from the filter paper using a paper puncher. Filter paper sections were incubated overnight at 4 °C in 300 μL of PBS buffer (0.1 M NaCl, 3 mM Na2HPO4 12H2O, 2 mM KCl, 1 mM KH2PO4, pH 7.2), then diluted in 500 μL of PBS supplemented with 0.05% Tween 20 and 30 g/L skim milk (Difco™), briefly vortexed, and centrifuged at 2000 rpm for 5 min to remove residual paper particles. The volumes of elution were adapted to mimic serum obtained from blood with a hematocrit value of 40%.

ELISA screening for CHIKV-specific IgG antibodies was performed from the filter paper eluates as described above. Preliminary testing of 50 filter paper/serum pairs confirmed that the thresholds determined the sera were applicable to filter paper eluates.

The quality of IgG detection in filter paper absorbed was performed using 144 paper/serum pairs. Briefly, a blind procedure was established for testing both serology techniques taking sera as controls, which enabled to calculate the Kappa coefficient, specificity and sensitivity of ELISA from filter paper eluates.

The Kappa coefficient was 0.90 (95% confidence interval 0.82–0.97). After excluding the grey zone results (five sera and one filter paper eluate), the calculated sensitivity of the filter paper ELISA technique was 97.9% (95% confidence interval 88.7–99.9) and the specificity was 100% (95% confidence interval 96.0–100.0).

**Additional reference**

1. **Grivard P, *et al*.** Molecular and serological diagnosis of chikungunya virus infection. *Pathologie et Biologie* 2007; **55**: 490-494.

**Supplemental file 12**

**Appendix 5. Description of meteorological data.**

Entomological studies on *Ae albopictus* suggest that temperature correlates with larval breeding sites [vii], rainfalls with adult population density [viii], and that drought can trigger outbreaks [vii]. Thus, three climate parameters were used: (1) average, minimal and maximal daily temperatures (°C), (2) daily precipitations (mm), and (3) outgoing long-wave radiations (W/m^2^). The latter three conditions were attempted to infer periods of hot temperature, moisture and drought. These factors are deemed to promote immature development, survival, longevity, fecundity, and gonotrophic cycles [ix]), adult mosquito pullulate (vii), or inadequate water storage behaviours associated with infection [viii]. Of note, relative humidity was not used as there was no rationale at that time that this parameter may drive *Ae albopictus* populations [x].

**Additional references**

1. **Chretien JP, *et al*.** Drought-associated chikungunya emergence along coastal east Africa. *American Journal of Tropical Medicine and Hygiene* 2007; **76**: 405-407.
2. **Serpa LL, *et al****.* Seasonal variation of Aedes aegypti and Aedes albopictus in a city of Southeastern Brazil. *Revista de Saude Publica*. 2006; **40**: 1101-1105.
3. **Delatte H, *et al.*** Influence of temperature on immature development, survival, longevity, fecundity, and gonotrophic cycles of Aedes albopictus, vector of chikungunya and dengue in the Indian Ocean. *Journal of Medical Entomology*. 2009: **46**: 33-41.
4. **Reiskind MH, Louinibos LP.** Effects of intraspecific larval competition on adult longevity in the mosquitoes Aedes aegypti and Aedes albopictus. Medical and Veterinary Entomology 2009; **23**: 62–68.

**Supplemental file 13**

**Appendix 6. Population attributable fraction and its 95% confidence interval.**

PAF= P(E+/D+) ×(RR-1) (1);

RR

combined PAF= 1-(1-PAF_1_) ×(1-PAF_2_) ×(1-PAF_3_)…(2)

95% CI for each determinant PAF were generated using the following formula:

Var (Ĥ)= Var [ln(1-PAF)]= _PAF^2^ ×[__Ṽ__ + ____2_____ +__A_0+____] (3) [xi].

(1-PAF_)_^2^ (ŘŘ-1)^2^ A_1+_×(ŘŘ-1) A_1+_ ×M_1_

*P: the proportion of exposed in the infected subjects; RR: relative risk for the exposure of interest; Ṽ: the variance estimator for the logarithm of the average relative risk ŘŘ; A_1+_: the number of exposed in the infected subjects; A_0+_: the number of unexposed in infected subjects; M_1+_: the total of infected subjects.*

**Additional references**

1. **Greenland S.** Application of stratified analysis methods. In Modern Epidemiology. 3^rd^ edition. Ed: Lippincott Williams & Wilkins, Philadelphia, USA. 2008; pp 283-302.

| Supplemental file 14  Appendix 7. STROBE Checklist of items that should be included in reporting of *cross-sectional studies* | | | | |
| --- | --- | --- | --- | --- |
|  | **Item No** | **Recommendation** | **Fulfilled** | **cf.** |
| **Title and abstract** | 1 | (*a*) Indicate the study’s design with a commonly used term in the title or the abstract | × | P1 |
|  |  | (*b*) Provide in the abstract an informative and balanced summary of what was done and what was found | × | P3 |
| Introduction | | |  |  |
| Background/rationale | 2 | Explain the scientific background and rationale for the investigation being reported | × | P4-P6  L56-L95 |
| Objectives | 3 | State specific objectives, including any prespecified hypotheses | × | P5  L96-L100 |
| Methods | | |  |  |
| Study design | 4 | Present key elements of study design early in the paper | × | P6  L102-L103 |
| Setting | 5 | Describe the setting, locations, and relevant dates, including periods of recruitment, exposure, follow-up, and data collection | × | P6, L104  Appendix 1 |
| Participants | 6 | (*a*) Give the eligibility criteria, and the sources and methods of selection of participants. Describe methods of follow-up | × | P6  L107-120 |
|  |  | (*b*) For matched studies, give matching criteria and number of exposed and unexposed | N.A | - |
| Variables | 7 | Clearly define all outcomes, exposures, predictors, potential confounders, and effect modifiers. Give diagnostic criteria, if applicable | × | P6-P7  L121-L149  Appendix 2-5 |
| Data sources/ measurement | 8* | For each variable of interest, give sources of data and details of methods of assessment (measurement). Describe comparability of assessment methods if there is more than one group | × | P6-P7  L121-L149  Appendix 2-5 |
| Bias | 9 | Describe any efforts to address potential sources of bias | × | P6-P8  L117-L20  L151-L163 |
| Study size | 10 | Explain how the study size was arrived at | × | P6  L106-L108 |
| Quantitative variables | 11 | Explain how quantitative variables were handled in the analyses. If applicable, describe which groupings were chosen and why | × | Appendix 2-4  P8  L169-L170 |
| Statistical methods | 12 | (*a*) Describe all statistical methods, including those used to control for confounding | × | P8-P9  L150-L180  Appendix 6 |
|  |  | (*b*) Describe any methods used to examine subgroups and interactions | × | P8  L166-L167 |
|  |  | (*c*) Explain how missing data were addressed | × | P9  L180 |
|  |  | (*d*) If applicable, explain how loss to follow-up was addressed | N.A | - |
|  |  | (*e*) Describe any sensitivity analyses | N.A | - |
| Results | | |  |  |
| Participants | 13* | (a) Report numbers of individuals at each stage of study—eg numbers potentially eligible, examined for eligibility, confirmed eligible, included in the study, completing follow-up, and analysed | × | Flow chart Fig.1 |
|  |  | (b) Give reasons for non-participation at each stage | × | Fig.1 |
|  |  | (c) Consider use of a flow diagram | × | Fig.1 |
| Descriptive data | 14* | (a) Give characteristics of study participants (eg demographic, clinical, social) and information on exposures and potential confounders  *Data are population-based estimates and the study was not conceived for assessing detailed demographic, clinical, social characteristics* | × | Page 10  L191-L199  Table 1 |
|  |  | (b) Indicate number of participants with missing data for each variable of interest  *Data are population-based estimates and missing observations or observations with missing data do not hamper the figures given for the community, which are straightened on population structure* | N.A | - |
|  |  | (c) Summarise follow-up time (eg, average and total amount) | N.A | - |
| Outcome data | 15* | Report numbers of outcome events or summary measures over time  *Numbers are population-based percentages, effect measures (prevalence proportion ratios, incidence rate ratios, odds ratios)* | × | Table 2  Table 3 |
| Main results | 16 | (*a*) Give unadjusted estimates and, if applicable, confounder-adjusted estimates and their precision (eg, 95% confidence interval). Make clear which confounders were adjusted for and why they were included | × | Tables 1-3, S2, S4,  S5, S8 |
|  |  | (*b*) Report category boundaries when continuous variables were categorised | Not done | -  Table S6 |
|  |  | (*c*) If relevant, consider translating estimates of relative risk into absolute risk for a meaningful time period | N.A | - |
| Other analyses | 17 | Report other analyses done—eg analyses of subgroups and interactions, and sensitivity analyses | × | Table S3  Table S6 |
| Discussion | | |  |  |
| Key results | 18 | Summarise key results with reference to study objectives | × | P15  L294-L317 |
| Limitations | 19 | Discuss limitations of the study, taking into account sources of potential bias or imprecision. Discuss both direction and magnitude of any potential bias | × | P19-P20  L402-L423 |
| Interpretation | 20 | Give a cautious overall interpretation of results considering objectives, limitations, multiplicity of analyses, results from similar studies, and other relevant evidence | × | P16-P19  L318-L401 |
| Generalisability | 21 | Discuss the generalisability (external validity) of the study results | × | P20  L424-L428 |
| Other information | | |  |  |
| Funding | 22 | Give the source of funding and the role of the funders for the present study and, if applicable, for the original study on which the present article is based | × | P9  L182-L189 |
| *Give information separately for exposed and unexposed groups. | | | | |
| **Note:** An Explanation and Elaboration article discusses each checklist item and gives methodological background and published examples of transparent reporting. The STROBE checklist is best used in conjunction with this article (freely available on the Web sites of PLoS Medicine at http://www.plosmedicine.org/, Annals of Internal Medicine at http://www.annals.org/, and Epidemiology at http://www.epidem.com/). Information on the STROBE Initiative is available at http://www.strobe-statement.org. | | | | |

**Supplemental file 15**

**Appendix 8. SEROCHIK Study Group investigators, collaborators and contributors ^‡^**

***Agence Régionale de Santé, Saint Denis, Reunion, France:*** Florence Caliez, Christine Catteau, Philippe Renault ; ***Centre de Coopération Internationale en Recherche Agronomique pour le Développement (CIRAD), Saint Denis, Reunion, France***: Eric Cardinale, PhD, Coralie Foray, DVM ; ***CIRAD, Saint Pierre, France****:* Frédéric Chiroleu, PhD*;* ***Centre Hospitalier Universitaire (CHU), Bordeaux, France***: Alexandre Duvignaud, MD ; ***CHU de la Réunion, Saint Pierre, Reunion, France***: Antoine Bertolotti, MD, Philippe Grivard, MD, Karin Le Roux, PhD, Patrice Poubeau, MD ; ***Cellule de Coordination des Recherches sur la Dengue et le Chikungunya INSERM-UPMC-EHESP, France***: Gilles Aumont, PhD, Véronique Boisson, MD, Antoine Flahault, PhD (coordinator), Sophie Journeaux, MD, Didier Fontenille, PhD, Xavier De Lamballerie, PhD, Vincent Lotteau, PhD, Christophe Paupy, PhD, Marie-Anne Sanquer, MD, Michel Setbon, PhD; ***Cellule d’Intervention Régionale de l’océan Indien (CIRE-OI), Saint Denis, Reunion, France***: Jean-Louis Solet, Eng ; ***INSERM Centre d’Investigation Clinique (CIE2), Saint Pierre, France***: Fabrice Bègue, Brigitte Bonal, Liliane Cotte, Cécile Dalban, Anne-Karen Le Pors, Annie Naty, Nadège Naty, Corinne Mussard, Joëlle Perrault, Sylvaine Porcherat ; ***INSERM Centre d’Investigation Clinique (CIC1410), Saint Pierre, France***: Karim Boussaïd, Olivier Rollot, Christophe Valingot ; ***INSEE, Saint Denis, Reunion, France***: Claude Parain ; ***Union Régionale des Professionnels de Santé / MEDOCEAN***: Philippe de Chazournes.
